# Folic Acid Oversupplementation Alters Global DNA Methylation and Circadian Clock Gene Expression in FaDu Head and Neck Squamous Cell Carcinoma Cells: An Exploratory Study

**DOI:** 10.3390/nu18193181

**Published:** 2026-09-26

**Authors:** Negin Faramarzi Garous, Liubov Lobanova, Silvana Papagerakis, Jessica R. L. Lieffers, Raed S. Said, Petros Papagerakis

**Affiliations:** 1 College of Pharmacy and Nutrition, University of Saskatchewan, Saskatoon, SK S7N 5E5, Canada; 2 Department of Anatomy, Physiology and Pharmacology, College of Medicine, University of Saskatchewan, Saskatoon, SK S7N 5E5, Canada; 3 Department of Ophthalmology and Otorhinolaryngology–Head and Neck Surgery, Faculty of Medicine, Université Laval, Quebec City, QC G1V 0A6, Canada; 4 ARThrite Research Center, Université Laval, Quebec City, QC G1V 4G2, Canada; 5 Department of Anatomy, College of Medicine and Health Sciences (CMHS), United Arab Emirates University (UAEU), Al Ain P.O. Box 15551, Abu Dhabi, United Arab Emirates; 6 Melbourne Dental School, University of Melbourne, Melbourne, VIC 3010, Australia

**Keywords:** folic acid, DNA methylation, circadian clock genes, head and neck squamous cell carcinoma (HNSCC), epigenetic regulation

## Abstract

Background/Objectives: Folic acid (FA), the synthetic form of folate (vitamin B9), is a vitamin involved in one-carbon metabolism, DNA synthesis, and DNA methylation. Because folate metabolism influences methyl donor availability, FA availability may affect circadian clock gene regulation through methylation-related or other metabolic mechanisms in head and neck squamous cell carcinoma (HNSCC). This exploratory study aims to determine whether FA oversupplementation influences cell viability and alters global DNA methylation, as well as the expression and methylation status of circadian clock genes in HNSCC cells. Methods: FaDu HNSCC cells were treated with 20 or 80 μM FA and compared with a no-added-FA (NF) reference condition. Cell viability was assessed using a CCK-8 assay after 48 and 72 h. For methylation and gene expression analyses, cells were synchronized with forskolin and treated with FA (20 or 80 μM) for 7 days. Global DNA methylation was measured using a 5-methylcytosine colorimetric assay. Following the NF and 80 μM FA treatment, cytosine-phosphate-guanine (CpG) island methylation in the *BMAL1* and *PER2* promoters, RNA expression, and protein levels were analyzed by bisulfite conversion followed by Sanger sequencing, qPCR, and ELISA. Results: FA treatment significantly reduced cell viability at 48 h (20 μM, *p* < 0.05; 80 μM, *p* < 0.01) and 72 h (both concentrations *p* < 0.01). Global DNA methylation was modestly reduced, reaching statistical significance only in the 80 μM FA group (*p* < 0.05). No qualitative differences were observed in the promoter methylation of the analyzed CpG regions of *BMAL1* and *PER2* following 80 μM FA treatment. However, *BMAL1* and *PER2* mRNA expressions were significantly increased (*p* < 0.05), while protein levels showed non-significant upward trends following 80 μM FA treatment. Conclusions: These exploratory findings suggest that the observed transcriptional changes are unlikely to be explained by methylation changes within the analyzed promoter regions of *BMAL1* and *PER2*. The mechanisms underlying these changes remain unclear and require further investigation.

## 1. Introduction

Folic acid (FA), the synthetic form of folate (vitamin B9), is an essential micronutrient involved in one-carbon metabolism [1]. It supports nucleotide biosynthesis, DNA synthesis and repair, and DNA methylation, thereby contributing to genomic stability and oral tissue integrity [1,2,3]. Folate is also critical for normal fetal development; its deficiency during pregnancy has been strongly associated with neural tube defects and impaired embryonic development [1]. Through one-carbon metabolism, folate contributes to the generation of S-adenosylmethionine (SAM), the universal methyl donor required for DNA and histone methylation [4,5]. Adequate folate availability is therefore critical for cellular homeostasis, whereas deficiency has been associated with oral mucosal disorders and impaired genomic stability [2,6,7].

Folate deficiency promotes uracil misincorporation, chromosomal instability, and aberrant methylation patterns that increase cancer susceptibility [8,9,10]. Consistently, folate-mediated one-carbon metabolism influences cancer-related pathways through epigenetic mechanisms, including DNA methylation changes which affect tumor suppressor genes and oncogenic signaling [2,11]. Because DNA methylation depends on methyl donors generated through one-carbon metabolism, folate availability represents a key metabolic determinant of epigenetic regulation and cancer-associated gene expression programs [1,2].

Circadian regulation coordinates fundamental cellular processes such as proliferation, apoptosis, DNA repair, and metabolism, and its disruption is increasingly recognized as a contributor to tumorigenesis [12,13]. At the molecular level, *CLOCK* and *BMAL1* activate rhythmic transcription of *PER* and *CRY* genes, whereas PER and CRY proteins repress *CLOCK/BMAL1* activity, forming a transcription–translation feedback loop that maintains circadian homeostasis [14,15].

Core clock genes, including *PER1* and *PER2*, are frequently described as tumor-suppressive regulators, and their reduced expression or promoter hypermethylation has been associated with malignant progression and genomic instability [16,17]. In oral squamous cell carcinoma (OSCC) and head and neck squamous cell carcinoma (HNSCC), circadian disruption has been linked to increased tumor aggressiveness, altered cell-cycle regulation, and unfavorable clinical outcomes [18,19,20]. However, the functional consequences of these alterations appear to be context dependent, highlighting the need to better understand factors regulating clock gene expression in HNSCC.

Emerging evidence suggests a mechanistic intersection between folate metabolism and circadian regulation. Experimental studies find that folate deficiency reduces circadian rhythm amplitude and alters clock gene expression, supporting a role for folate-dependent epigenetic mechanisms in circadian control [21,22]. These observations raise the possibility that metabolic availability of methyl donors may influence circadian gene regulation through epigenetic remodeling, an interaction that remains poorly characterized in cancer.

HNSCC remains a common and aggressive malignancy, frequently diagnosed at advanced stages and characterized by high metastatic potential and unpredictable therapeutic responses [23,24]. Although circadian dysregulation is widely documented in HNSCC and OSCC, expression patterns of clock genes are not uniformly altered. Reduced expression or epigenetic silencing of *PER1*, *PER2*, and *BMAL1* has been frequently reported, yet context-dependent variability related to tumor stage, anatomical site, and molecular subtype is increasingly recognized [16,18]. Alterations in circadian clock genes, including *BMAL1*, *PER1*, and *PER2*, have been reported in HNSCC, OSCC, and other cancers, with their expression associated with tumor-related cellular processes and clinical outcomes [25,26,27,28,29]. However, the biological roles of these genes appear to vary across cancer types and cellular contexts [16,18]. Therefore, further investigation of factors that may influence circadian gene regulation in HNSCC is warranted.

Despite growing evidence linking folate metabolism, epigenetic mechanisms, and circadian regulation to cancer progression, whether FA availability influences circadian clock gene regulation in HNSCC remains unknown. Therefore, we hypothesized that FA oversupplementation may modulate circadian gene expression via methylation-dependent mechanisms, thereby influencing cellular behavior. To our knowledge, this study is the first to explore within a unified experimental framework whether FA oversupplementation modulates circadian clock gene expression together with associated methylation changes in HNSCC cells. Accordingly, this study aims to determine whether FA oversupplementation influences cell viability and alters global DNA methylation, as well as the expression and methylation status of circadian clock genes in HNSCC cells. Overall, this exploratory work provides baseline knowledge of circadian gene regulation under FA exposure and establishes a foundation for future investigations into the time- and dose-dependent effects of FA on HNSCC epigenetics, disease progression, and therapeutic response.

## 2. Materials and Methods

### 2.1. Cell Culture and Folic Acid Treatment

The human hypopharyngeal squamous cell carcinoma cell line FaDu (HTB-43; ATCC, Manassas, VA, USA) was used as a model of HNSCC. Cells at passages 11–13 were maintained in FA-free RPMI-1640 medium (Cat. No. 27016-021, Gibco, Thermo Fisher Scientific, Waltham, MA, USA) supplemented with 10% fetal bovine serum (FBS; Cat. No. 12483020, Invitrogen, Thermo Fisher Scientific, Waltham, MA, USA), 1× penicillin–streptomycin (Cat. No. SV30010, Thermo Fisher Scientific, Waltham, MA, USA), and 1 mM sodium pyruvate (Cat. No. SH30239.01, HyClone, Cytiva, Marlborough, MA, USA).

For the 7-day FA exposure experiments used for subsequent molecular and epigenetic analyses, cells were initially cultured in T75 flasks until reaching approximately 70% confluency. Cells were then seeded into 6-well plates at a density of 1 × 10^3^ cells/mL, with 2 mL of cell suspension added per well, corresponding to 2 × 10^3^ cells/well. Circadian synchronization was performed using a two-step serum starvation and forskolin treatment protocol. Cells were first serum-starved for 24 h in FA-free RPMI-1640 medium containing 1× penicillin–streptomycin and 1 mM sodium pyruvate with no FBS. Cells were subsequently treated with 15 µM forskolin (Cat. No. F3917, Sigma-Aldrich, St. Louis, MO, USA) for 2 h in the same serum-free, FA-free medium.

Following synchronization, cells were washed twice with phosphate-buffered saline (PBS; Cat. No. SH30256.01, HyClone, Cytiva, Marlborough, MA, USA) and cultured for 7 days in supplemented FA-free RPMI-1640 medium without added FA (no-added-FA [NF] reference condition) or in the same medium supplemented with 20 µM or 80 µM FA (Thermo Fisher Scientific, Waltham, MA, USA). Treatment media for all three groups were replaced every 24 h throughout the 7-day exposure period. The 20 and 80 µM FA concentrations were selected as relatively high experimental concentrations based on previous studies investigating FA exposure in cancer cell models [30,31].

### 2.2. Cell Viability Assessment-CCK-8 Assay

Cell viability was assessed in experiments conducted separately from the 7-day FA exposure experiments described above. FaDu cells within the same passage range were seeded into 96-well plates at a density of 1 × 10^3^ cells/well and cultured in the basal medium described in Section 2.1. Cells were exposed to the supplemented FA-free RPMI-1640 medium described in Section 2.1 without added FA (NF) or to the same medium supplemented with 20 µM or 80 µM FA. Forskolin synchronization and serum starvation were not performed for the CCK-8 cell viability experiments.

Cell viability was assessed after 48 and 72 h of FA exposure using the CCK-8 Cell Viability Assay Kit (Cat. No. CK04-01, Dojindo Laboratories, Kumamoto, Japan) according to the manufacturer’s instructions. Briefly, 10 µL of CCK-8 solution was added to each well, followed by incubation for 1 h at 37 °C protected from light. Absorbance was then measured at 450 nm using a Varioskan LUX Multimode Microplate Reader (Thermo Fisher Scientific, Waltham, MA, USA). Cell viability was expressed as a percentage relative to the NF reference condition, which was normalized to 100%.

Cell viability experiments were performed using eight independent biological replicates per treatment condition (*n* = 8), with each biological replicate measured in duplicate technical wells. The mean background absorbance of the blank wells was subtracted from each measurement. Afterward, the two technical replicate values were averaged to obtain one value for each biological replicate. Cell viability was normalized to the mean value of the no-added-folic-acid (NF) reference group, which was set to 100%, and results were expressed as percentage viability relative to NF. Statistical analyses were performed using the eight independent biological replicate values per group. The 48-h and 72-h experiments were analyzed separately. At each time point, differences among the NF, 20 µM FA (FA-20 µM), and 80 µM FA (FA-80 µM) groups were assessed using ordinary one-way ANOVA followed by Dunnett’s multiple-comparisons test, with NF specified as the reference group. Statistical analyses were performed using GraphPad version 11.0.2 (GraphPad Software, Boston, MA, USA). Individual optical density (OD) measurements obtained using a Varioskan LUX Multimode Microplate Reader (Thermo Fisher Scientific, Waltham, MA, USA) for the biological replicates and their technical duplicates, together with the corresponding blank measurements, are provided in Supplementary Tables S1 and S2.

### 2.3. DNA, RNA, and Protein Extraction of the Treated Cells After 7 Days

After 7 days of FA and NF treatment, cells were washed twice with 1X phosphate-buffered saline (PBS; Cat. No. SH30256.01, HyClone, Cytiva, Marlborough, MA, USA) and detached using 0.025% trypsin–EDTA (Cat. No. SH30042.02, HyClone, Cytiva, Marlborough, MA, USA). DNA, RNA, and protein were subsequently isolated using the DNA/RNA/Protein Purification Plus Kit (Cat. No. 47700, Norgen Biotek Corp., Thorold, ON, Canada) following the manufacturer’s protocol. The concentration and purity of the isolated biomolecules were measured using NanoDrop ND-2000 spectrophotometer (Thermo Fisher Scientific, Wilmington, DE, USA).

### 2.4. Folic Acid Effects on Global and Targeted DNA Methylation

#### 2.4.1. Global DNA Methylation Assessment Following Folic Acid Treatment

Global DNA methylation (5-methylcytosine, 5-mC) was quantified using the Global DNA Methylation Assay Kit (5 Methyl Cytosine, Colorimetric) (Cat. No. ab233486, Abcam, Cambridge, UK) according to the manufacturer’s instructions. A total of 100 ng of genomic DNA isolated from FaDu cells treated with FA (20 µM or 80 µM) or NF control was used for each reaction. Briefly, DNA was immobilized in assay strip wells, and methylated cytosines were detected using a 5-mC-specific capture and detection antibody system with colorimetric signal development. Absorbance was measured at 450 nm using a Varioskan LUX Multimode Microplate Reader (Thermo Fisher Scientific, Waltham, MA, USA) following termination of the enzymatic reaction. Standard dilutions were prepared for 0%, 0.1%, 0.2%, 0.5%, 1%, 2%, and 5% 5-mC. Standard curves were generated by plotting Optical Density (OD) values at 450 nm against the percentage of 5-mC at each concentration point. Linear regression analysis was performed using Microsoft Excel version 2608 (Microsoft Inc, Redmond, WA, USA) to determine the slope (OD/1%) of the standard curve. According to the kit manufacturer, at least four concentration points, including the 0 point, were needed for obtaining the optimal slope. The analysis for FA-20 µM vs. NF and FA-80 µM vs. NF were performed separately. For NF vs. FA-20 µM, standards containing 0%, 0.1%, 0.2%, 1% 5-mC were used to determine the optimal slope, whereas for NF vs. FA-80 µM, standards containing 0%, 0.1%, 0.2%, and 0.5% 5mC were used. Those standard curves are presented in Supplementary Figures S1 and S2.

For sample calculations, the mean OD of the blank wells was subtracted from each sample OD measurement. The percentage of 5-mC in each sample was then calculated using the blank-corrected OD, the slope obtained from the corresponding standard curve, and the DNA input amount (100 ng), according to the manufacturer’s calculation method. Each biological replicate was analyzed in duplicate, and the technical duplicates were averaged to obtain one 5-mC value for each biological replicate. Three independent biological replicates were analyzed per treatment condition (*n* = 3), and the results are presented as mean ± SD. The individual OD measurements for the biological replicates and their technical duplicates, together with the corresponding blank measurements and duplicate OD measurements of the standards used to generate the standard curves, are provided in Supplementary Tables S3–S6.

#### 2.4.2. Targeted DNA Methylation Assessment Following Folic Acid Treatment

Targeted DNA methylation analysis was performed on genomic DNA isolated from cells treated with 80 µM-FA and NF controls using sodium bisulfite conversion, followed by Polymerase Chain Reaction (PCR) amplification and direct Sanger sequencing. This approach was used to assess cytosine-phosphate-guanine (CpG) island methylation patterns at selected loci of interest. All processes are explained below.

##### CpG Island Identification for Clock Genes

CpG islands associated with the *BMAL1* and *PER2* genes were identified and analyzed in cells treated with FA-80 µM vs. NF control samples. For *BMAL1*, CpG sites and CpG island boundaries within the promoter region were identified using the UCSC Genome Browser (GRCh37/hg19) (https://genome.ucsc.edu, accessed on 5 August 2026), following the criteria and genomic coordinates described by Peng et al. (2019) [32]. For *PER2*, CpG islands were predicted using MethPrimer (http://www.urogene.org/methprimer, accessed on 5 December 2022) according to Li and Dahiya (2002) [33] and Boudin et al. (2015) [34]. Two CpG islands located within the promoter and 5′ untranslated region (5′ UTR) were selected for targeted bisulfite sequencing. In addition, a CpG-rich region within the *PER2* coding region was included as a control region, as it is predicted to be constitutively methylated and thus serves as a reference for fully methylated CpG sites.

##### DNA Bisulfite Treatment

DNA isolated from FaDu cells in the FA-80 µM and NF groups, as previously described, was bisulfite-converted using the EZ DNA Methylation Kit (Cat. No. D5001 and D5002, Zymo Research, Irvine, CA, USA) according to the manufacturer’s protocol. Sodium bisulfite treatment converts unmethylated cytosines to uracil, which are subsequently amplified as thymine during PCR, whereas 5-methylcytosines remain unconverted, thereby enabling discrimination between methylated and unmethylated CpG sites at single-base resolution.

##### Primer Design for Targeted Bisulfite DNA Methylation Analysis

Target genomic regions (CpG islands) were converted to bisulfite-modified sequences in silico using Bisulfite Primer Seeker (Zymo Research, Irvine, CA, USA). Primers specific for bisulfite-converted DNA were designed using the ZYMO Bisulfite Primer Design Tool. The primer sequences and targeted regions are listed in Supplementary Tables S7 and S8.

##### Targeted Bisulfite DNA PCR

Bisulfite PCR amplification was performed using gene-specific primers described previously. Thermal cycling was carried out under the following conditions: an initial denaturation at 94 °C for 2 min, followed by 40 cycles of denaturation at 94 °C for 30 s, annealing at 55–60 °C for 30 s, and extension at 72 °C for 30 s, with a final extension at 72 °C for 5 min. PCR products were verified by agarose gel electrophoresis and prepared for Sanger sequencing according to standard protocols.

##### Bisulfite Sanger Sequencing and Data Analysis

PCR amplicons generated from bisulfite-converted genomic DNA were subjected to Sanger sequencing. Raw ABI chromatogram files obtained from Sanger sequencing were imported into CodonCode Aligner v12.0.5 (CodonCode Corporation, Centerville, MA, USA) for sequence alignment and methylation analysis. All sequencing chromatograms were manually inspected to confirm high-quality signal intensity, accurate base calling, and correct alignment across the targeted regions.

For data interpretation, two reference sequence keys were used. The genomic key corresponded to the native, unconverted genomic DNA sequence. The bisulfite key consisted of an in-silico bisulfite-converted reference sequence in which all non-CpG cytosines were converted to thymine, whereas cytosines within CpG dinucleotides were retained as cytosine to account for potential methylation at CpG sites. Bisulfite-converted reference sequences were generated using the Zymo Research Bisulfite Primer Designer tool in accordance with standard bisulfite conversion rules. Sequencing reads were aligned to both the bisulfite-converted reference sequence and the genomic DNA key using CodonCode Aligner. Bisulfite conversion efficiency was assessed by verifying the complete conversion of non-CpG cytosines to thymine throughout the analyzed regions. Only sequencing reads demonstrating efficient conversion and high-quality chromatograms were included in downstream analyses.

CpG methylation status was evaluated qualitatively based on chromatogram peak patterns at CpG positions. Cytosine (C) peaks were interpreted as methylated CpG sites, thymine (T) peaks as unmethylated CpG sites, and overlapping cytosine/thymine (C/T) peaks as indicative of heterogeneous methylation within the amplified DNA population. Therefore, CpG methylation patterns were assessed descriptively, evaluated for consistency across biological replicates, and compared between the FA-80 µM treatment group and NF control samples.

### 2.5. Real-Time Quantitative Polymerase Chain Reaction (RT-qPCR)

RNA isolated from FaDu cells in the FA-80 µM and NF groups, as previously described were used for Complementary DNA (cDNA) synthesis. cDNA was synthesized from 400 ng of total RNA using the High-Capacity cDNA Reverse Transcription Kit (Cat. No. 4368814, Lot No. 01152470, Applied Biosystems™, Thermo Fisher Scientific, Waltham, MA, USA) according to the manufacturer’s protocol. Quantitative real-time PCR (qPCR) was conducted using the Power SYBR™ Green Master Mix (Cat. No. 4367659, Applied Biosystems™, Thermo Fisher Scientific, Waltham, MA, USA). Relative mRNA expression levels of *BMAL1*, and *PER2* were determined using the 2^−ΔΔCt^ method, with *β2M* (Beta-2-microglobulin) as the endogenous reference gene. Primer sequences are listed in Supplementary Table S9. The individual cycle threshold (Ct) values for *BMAL1*, *PER2*, and *B2M* from the biological replicates and their technical duplicates are provided in Supplementary Table S10.

### 2.6. Protein Assessment

Isolated proteins from the FA-80 µM treatment group and NF control samples, described previously, were used for PER2 and BMAL1 protein assessment using a Human PER2 ELISA kit (Cat. No. XPEH11019, XpressBio, Frederick, MD, USA) and a Human ARNTL/BMAL1 ELISA kit (Cat. No. XPEH6460, Xpress Biotech International, Frederick, MD, USA) according to the manufacturer instructions.

#### 2.6.1. ELISA Kit Process for PER2 Protein Assessment

Briefly, PER2 standards were prepared at concentrations of 10, 5, 2.5, 1.25, 0.625, 0.312, and 0 ng/mL. Samples were prepared at a total protein concentration of 2 mg/mL, within the manufacturer-recommended range of 1–3 mg/mL, and the assay was performed according to the manufacturer’s instructions. A total volume of 100 µL of each standard, sample, or blank was added to the appropriate wells. Three independent biological replicates were analyzed per condition (*n* = 3), with each biological replicate analyzed in duplicate. Following completion of the assay, absorbance (OD) was measured at 450 nm using a Varioskan LUX Multimode Microplate Reader (Thermo Fisher Scientific, Waltham, MA, USA).

For the calculation of PER2 protein concentrations, the mean blank OD_450_ value was subtracted from the OD_450_ values of the standards and samples. A four-parameter logistic standard curve was generated by plotting the mean blank-corrected absorbance of each standard against its corresponding concentration. Sample protein concentrations were determined from the standard curve, and technical duplicates were averaged to obtain one protein concentration for each biological replicate. The standard curve is presented in Supplementary Figure S3. The individual OD_450_ measurements for the biological replicates and their technical duplicates, together with the corresponding blank measurements and duplicate OD_450_ measurements of the standards used to generate the standard curve, are provided in Supplementary Tables S11 and S12.

#### 2.6.2. ELISA Kit Process for BMAL1 Protein Assessment

Briefly, BMAL1 protein levels were assessed using the same procedure described above for PER2, with BMAL1-specific standards prepared at concentrations of 1, 0.5, 0.25, 0.125, 0.0625, 0.0312, and 0 ng/mL. The corresponding standard curve is presented in Supplementary Figure S4, and the individual OD_450_ measurements for the biological replicates and their technical duplicates, together with the blank and standard measurements, are provided in Supplementary Tables S13 and S14.

### 2.7. Statistical Analysis

Statistical comparisons were performed between the no-added-folic-acid (NF) reference condition and FA-treated conditions (20 µM and/or 80 µM FA, as applicable to each experiment). Comparisons between two groups were performed using unpaired *t*-test. For experiments involving three groups (NF, FA-20 µM, and FA-80 µM), one-way analysis of variance (ANOVA) followed by Dunnett’s multiple-comparisons test was used to compare each FA-treated group with the NF reference condition. Statistical analyses were performed using GraphPad Prism version 11.1.2 (GraphPad Software, Boston, MA, USA). *p* values < 0.05 were considered statistically significant (*p* < 0.05; * *p* < 0.01). Data are presented as mean ± standard deviation (SD). Microsoft Excel was used for data organization and calculations, and graphical illustrations were generated using GraphPad Prism. 

## 3. Results

### 3.1. Effect of Folic Acid Treatment on FaDu Cell Viability

FaDu cell viability was evaluated following 48 and 72 h of treatment with 20 µM and 80 µM FA compared with the NF reference condition. At 48 h, one-way ANOVA showed a significant overall difference in cell viability among the treatment groups (F(2,21) = 7.34, *p* = 0.0038). Dunnett’s multiple-comparisons test showed that cell viability was significantly reduced following treatment with both FA-20 µM (*p* = 0.0128) and FA-80 µM (*p* = 0.0036) compared with NF. At 72 h, one-way ANOVA similarly showed a significant overall difference among the treatment groups (F (2,21) = 8.739, *p* = 0.0017). Cell viability was significantly reduced following treatment with both FA-20 µM (*p* = 0.0030) and FA-80 µM (*p* = 0.0031) compared with NF, as determined by Dunnett’s multiple-comparisons test (Figure 1).

### 3.2. Global DNA Methylation Levels

Global DNA methylation levels were assessed using a 5-methylcytosine colorimetric assay. Global DNA methylation analysis showed a statistically significant reduction in 5-mC levels following treatment with FA-80 µM compared with the NF reference condition (*p* = 0.0360), with 5-mC levels corresponding to 0.890-fold relative to NF. In contrast, treatment with FA-20 µM resulted in 0.949-fold 5-mC levels relative to NF, and the reduction was not statistically significant (*p* = 0.2050) (Figure 2). Data are presented as mean ± SD from three independent biological replicates per condition (*n* = 3), with each biological replicate analyzed in duplicate.

### 3.3. Impact of Folic Acid Treatment on Targeted DNA Methylation Patterns

The DNA methylation status of individual CpG sites within the promoter regions of *BMAL1* and *PER2*, as well as the 5′ untranslated region (UTR) of *PER2*, was qualitatively assessed in NF control cells and cells treated with 80-µM FA using direct bisulfite Sanger sequencing. CpG methylation status was determined from chromatogram peak patterns and classified as unmethylated (T), methylated (C), or heterogeneous (C/T). For *BMAL1*, 13 CpG sites located within the promoter region were analyzed. All analyzed CpG sites exhibited thymine-only signals following bisulfite conversion, indicating an unmethylated status in both NF and 80-µM FA-treated cells, with no qualitative differences observed between groups. For *PER2*, two CpG-rich regions were examined. In the 5′ UTR, all 16 analyzed CpG sites displayed thymine-only signals in both groups, consistent with an unmethylated status. Within the promoter-associated CpG island, 28 CpG sites were evaluated. One CpG site exhibited overlapping cytosine and thymine peaks, indicative of heterogeneous methylation, whereas the remaining CpG sites were unmethylated. The same qualitative methylation pattern was observed in NF controls and 80-µM FA-treated cells. As a control, a CpG-rich region within the coding region of *PER2* was also analyzed. Eight CpG sites were assessed, all of which exhibited cytosine-only signals, indicating methylated CpG sites in both groups. This observation is consistent with the genomic location of the analyzed region within the gene body, which is generally expected to exhibit higher levels of DNA methylation. The detection of unmethylated, heterogeneous, and methylated CpG patterns across the analyzed regions supported the reliability of the bisulfite conversion and Sanger sequencing workflow. Overall, no treatment-associated qualitative differences in CpG methylation patterns were detected between NF and 80-µM FA-treated cells across the analyzed loci. Detailed genomic positions and qualitative methylation calls are summarized in Figure 3. Representative Sanger sequencing chromatograms demonstrating methylated, heterogeneous, and unmethylated DNA methylation patterns are provided in the Supplementary Figure S5.

### 3.4. Folic Acid Upregulates BMAL1 and PER2 mRNA Expression

To evaluate the effects of FA treatment on the mRNA expression levels of the target clock genes, the expression of *BMAL1* and *PER2* were analyzed, with *B2M* used as the reference gene for normalization. Treatment with FA-80 µM resulted in a statistically significant upregulation of *BMAL1* expression compared with the NF reference condition (*p* = 0.0230), corresponding to a 4.5-fold increase relative to NF. Similarly, *PER2* expression was significantly upregulated following treatment with FA-80 µM compared with NF (*p* = 0.0371), corresponding to a 3-fold increase relative to NF (Figure 4). Data are presented as mean ± SD from three independent biological replicates per condition (*n* = 3), with each biological replicate analyzed in duplicate.

### 3.5. Effect of Folic Acid Treatment on BMAL1 and PER2 Protein Expression

To evaluate the effects of FA treatment on the protein levels of the target clock proteins, BMAL1 and PER2 protein levels were assessed using colorimetric ELISA assays. Treatment with FA-80 µM did not result in a statistically significant difference in BMAL1 protein levels compared with the NF reference condition (*p* = 0.9122). Similarly, PER2 protein levels were not significantly different following treatment with FA-80 µM compared with NF (*p* = 0.3244) (Figure 5). Data are presented as mean ± SD from three independent biological replicates per condition (*n* = 3), with each biological replicate analyzed in duplicate.

## 4. Discussion

The present exploratory study investigated cell viability, global DNA methylation, and circadian clock gene expression following FA oversupplementation in FaDu HNSCC cells. *BMAL1* and *PER2* promoter CpG methylation, mRNA expression, and protein levels were also evaluated. Because folate plays a central role in one-carbon metabolism and provides methyl groups for the synthesis of S-adenosylmethionine (SAM), the universal methyl donor for DNA methylation, alterations in folate availability have the potential to influence cellular DNA methylation. However, DNA methylation represents only one of several mechanisms regulating gene expression, and promoter methylation of specific genes is determined by multiple factors, including DNA methyltransferase activity, chromatin accessibility, transcription factor binding, and genomic context. Consequently, the relationship between folate availability and promoter methylation is highly context dependent. In the present study, FA exposure increased BMAL1 and PER2 mRNA expression and was associated with a reduction in global DNA methylation, whereas no detectable promoter methylation changes were observed within the analyzed CpG regions of either gene. Therefore, our findings do not support direct regulation of BMAL1 or PER2 expression through methylation changes within the analyzed promoter regions. The mechanisms underlying the observed changes in *BMAL1* and *PER2* mRNA expression remain unclear and require further investigation. These findings provide preliminary evidence that warrants further investigation of the relationship between folate availability and circadian regulation in HNSCC.

FA is used as a nutritional supplement and as an adjunct to cancer therapy, where its role in genetic and epigenetic regulation may contribute to dual effects in established neoplasms and support chemotherapy efficacy in colorectal cancer [35]. However, its potential effects in HNSCC remain less well understood, highlighting the need of investigating FA-related molecular changes in this cancer type. In our study, increasing FA concentrations reduced HNSCC cell viability, with both 20 and 80 µM FA significantly decreasing viability compared with NF conditions after 48 and 72 h. These observations are consistent with findings in colorectal cancer cell lines exposed to supraphysiological FA concentrations (10,000 ng/mL), where viability decreased in HT-29 cells but remained unchanged in SW480 cells, indicating a cell line–dependent response [35]. In contrast, a study in HepG2 cells (hepatocellular carcinoma cell line) reported that FA deficiency (no folic acid) reduced cell viability, whereas FA oversupplementation (100 µM) was associated with a higher number of viable cells, although this increase was not statistically significant [30]. Collectively, these findings suggest that the biological effects of FA are context-dependent and may vary according to cancer type, cellular context, FA concentration, and the metabolic and epigenetic state of the cells.

Consistent with the central role of folate in one-carbon metabolism, we next examined whether FA oversupplementation was associated with alterations in global DNA methylation. Based on 5-methylcytosine quantification, a significant reduction was observed in the FA-80 µM treatment group compared with the NF. A reduction trend was also observed in the FA-20 µM treatment group; however, this difference was not statistically significant. Previous studies have reported variable effects of FA on global DNA methylation (5-methylcytosine detection), highlighting the importance of cancer context and analytical approach. In colorectal cancer, Farias et al. (2015) demonstrated that high-dose FA reduced global DNA methylation in HCT116 and LS174T cells compared to NF, while no statistically significant changes were observed in SW480 cells, indicating a cell line–dependent response [36]. Additionally, Zsigrai et al. (2022) reported that global DNA methylation (LINE-1 methylation level detection) remained largely unchanged after FA treatment despite methylation alterations at specific genomic regions [35].

Together, these findings, including our data, suggest that the effects of altered folate availability on global DNA methylation vary according to cellular and experimental context. Because DNA methylation is catalyzed by DNA methyltransferases (DNMTs), which use SAM as the methyl donor, differences in folate availability may influence methylation indirectly through cellular metabolic regulation rather than through a direct effect of FA itself. The variability observed across studies further indicates that global DNA methylation responses depend on cellular background, metabolic state, experimental conditions, and the methodological approach used for methylation assessment.

Promoter DNA methylation is widely regarded as a key epigenetic mechanism linking metabolic state (through methyl-donor availability) to transcriptional regulation [11,36]. Unlike global DNA methylation, which reflects overall genomic methylation status, promoter methylation is regulated at individual loci through DNA methyltransferase activity, chromatin accessibility, transcription factor binding, and local genomic context. Consequently, changes in global DNA methylation do not necessarily result in detectable methylation changes at specific gene promoters.

To investigate whether methylation-related mechanisms were associated with changes in circadian gene expression following FA exposure, we evaluated both global DNA methylation and promoter methylation of *BMAL1* and *PER2*. Although FA exposure was associated with reduced global DNA methylation and increased BMAL1 and PER2 mRNA expression, no detectable promoter methylation changes were observed within the analyzed CpG regions. These findings suggest that the observed transcriptional changes are unlikely to result from methylation changes within the analyzed promoter CpG regions.

This observation described above is consistent with previous evidence indicating that promoter methylation can remain stable even under epigenetic modulation. For example, Hernández-Rosas et al. (2018) reported no detectable changes in *PER2* promoter methylation within the analyzed CpG regions following treatment with epigenetic modulators [37]. Together, these findings indicate that alterations in cellular metabolic or epigenetic conditions do not necessarily translate into promoter-specific methylation remodeling. Therefore, the mechanisms underlying the observed changes in BMAL1 and PER2 mRNA expression in FaDu cells remain unclear and cannot be explained by methylation changes within the analyzed promoter regions.

Consistent with the proposed role of circadian clock genes in tumor regulation, FA treatment in our study was associated with increased expression of key clock components. We detected statistically significant upregulation of *BMAL1* and *PER2* mRNA expression following FA-80 µM treatment in HNSCC cells. In this context, the observed transcriptional upregulation may reflect increased expression of circadian regulatory components, although functional circadian restoration cannot be inferred from single-time-point expression data. Similarly, promoter methylation was evaluated at a single experimental time point; therefore, temporal changes in DNA methylation associated with circadian regulation cannot be excluded. Notably, the biological roles of these genes are not uniform across tumor contexts [26,27,38]. Prior studies have demonstrated that core clock components such as *BMAL1* and *PER1* can exert divergent effects depending on cellular background and oncogenic signaling, including context-dependent associations with patient survival and therapeutic resistance [27,38]. Therefore, the implications of their increased expression in the present model should be interpreted within this cell-specific framework. This finding is consistent with reports describing circadian clock dysregulation in head and neck and oral cancers, frequently involving reduced expression of specific clock components associated with tumor progression and adverse clinical features [16,17,18].

Despite the changes observed at the mRNA level, BMAL1 and PER2 protein levels showed only a non-significant upward trend following FA treatment. The relationship between mRNA and protein abundance is influenced by multiple regulatory processes, including translational efficiency and post-translational regulation of protein stability and degradation [39]. Therefore, increased mRNA abundance does not necessarily result in a proportional increase in the corresponding protein. Under the experimental conditions examined, the response to FA exposure was more evident at the mRNA level; however, the biological significance of the observed protein trends remains uncertain and requires further investigation.

Taken together, these findings indicate that the observed changes in *BMAL1* and *PER2* mRNA expression were not accompanied by detectable methylation changes within the analyzed promoter regions. The mechanisms underlying these expression changes remain unclear, and additional functional studies are required to determine their biological significance in HNSCC cells.

Folate deficiency has been associated with megaloblastic anemia, cardiovascular and obstetrical complications, neuropsychiatric disorders, and malignancies [35], as well as oral mucosal disorders and impaired genomic stability [2,6,7]. Beyond its established role in deficiency states, folate demonstrates context-dependent effects in carcinogenesis and cancer therapy. While FA is used adjunctively in colorectal cancer to enhance fluoropyrimidine efficacy, treatment tolerance more broadly appears to vary according to folate status, with deficient status increasing toxicity during antifolate therapy and higher folate levels potentially exacerbating toxicity during fluoropyrimidine-based treatment [40]. In the context of HNSCC, where oral epithelial integrity and genomic stability are central to disease progression and treatment response, these interactions are particularly relevant. Although oncology-specific supplementation guidelines are not established, the Recommended Dietary Allowance (RDA) for the general Canadian population advises 400 µg dietary folate equivalents (DFE) per day for adults aged ≥19 years, 600 µg per day during pregnancy and 500 µg per day during lactation [41,42]. Oral health professionals should continue to follow current dietary folate recommendations, as our study findings do not support changes to existing recommendations. Rather, as this was an exploratory study, the findings highlight the need for further research on the effects of FA in HNSCC.

HNSCC is a biologically and histologically heterogeneous disease, with variation across anatomical sites, molecular subtypes, and histological variants. Rare aggressive variants, such as adenosquamous carcinoma of the oral cavity, further illustrate this heterogeneity and may exhibit distinct clinical behavior [43]. HPV status also represents a major determinant of biological variation in HNSCC [44]. In this exploratory study, we used the HPV-negative FaDu cell line; however, future work should include HPV-positive HNSCC models (e.g., FaDu-derived cells that express HPV, such as 2A3) to better reflect this diversity. High-risk HPV increases cancer risk primarily through the activity of viral oncogenes, which disrupt key tumor suppressor pathways and interfere with normal cell cycle regulation [45,46]. At the same time, HPV-positive tumors behave differently and often respond better to treatment, with more favorable clinical outcomes than HPV-negative tumors [47]. Clinical data further show that HPV status correlates strongly with survival and associated gene expression patterns, whereas traditional risk factors such as tobacco and alcohol use, as well as tumor stage, show weaker or no clear association with these molecular networks [44].

FA plays an important role in one-carbon metabolism and DNA methylation. Observational evidence suggests an inverse association between dietary folate intake and HPV infection risk [48]. Because epigenetic regulation, including DNA methylation, contributes to circadian gene regulation, folate levels may indirectly influence pathways involved in cell cycle control, DNA repair, and tumor behavior. HPV-related changes in host gene regulation may further interact with these circadian mechanisms. Studying FA in HPV-positive models may help clarify how folate metabolism interacts with HPV-related pathways, circadian regulation, and treatment response.

This exploratory study provides an integrated analysis of cellular responses to FA exposure in HNSCC, combining cell viability assays, global and promoter-specific DNA methylation profiling, and RNA/protein expression analyses in the FaDu cell model. By focusing on key clock genes (*BMAL1*, *PER2*) within the context of FA exposure, it provides preliminary information on changes in DNA methylation and circadian gene expression associated with FA exposure in FaDu HNSCC cells.

The use of multiple FA concentrations and a defined exposure period allowed comparison of cellular responses across FA exposure conditions, while comparison with previous colorectal cancer studies provides additional context for the observed findings [36]. This study was intended as a pilot investigation using a cell line that was already undergoing comprehensive circadian analysis (Kahn et al., under review). Furthermore, the discrepancy between mRNA and protein expression indicates that additional studies are needed to better understand the regulation of circadian gene expression following FA exposure.

FA exposure was associated with reduced global DNA methylation and increased BMAL1 and PER2 mRNA expression; however, no significant methylation changes were detected within the analyzed promoter regions of either gene. Therefore, the relationship between the observed global DNA methylation changes and BMAL1 and PER2 expression remains unclear. The present findings do not demonstrate that the changes in clock gene expression were mediated by DNA methylation, and other regulatory mechanisms were not examined in this study. Further mechanistic studies are needed to determine how FA exposure influences BMAL1 and PER2 expression in HNSCC cells.

All experimental groups were maintained under the same basal culture conditions, including the same FA-free medium and FBS concentration, with FA added at 0, 20, or 80 µM. Thus, the observed differences represent cellular responses across conditions of no-added FA and high FA exposure. However, because the folate content of the FBS was not quantified and a physiological folate control was not included, the NF condition cannot be considered completely folate-free or physiologically representative. Therefore, the observed effects cannot be attributed exclusively to FA oversupplementation and may also reflect cellular responses associated with the no-added-FA condition.

## 5. Conclusions

This exploratory study showed that FA exposure was associated with reduced cell viability and global DNA methylation in FaDu HNSCC cells, with the reduction in global DNA methylation reaching significance at FA-80 µM. At the same concentration, *BMAL1* and *PER2* mRNA expression was significantly increased, whereas protein levels showed only non-significant upward trends. No detectable methylation changes were observed within the analyzed promoter regions of *BMAL1* or *PER2*, indicating that the observed transcriptional changes cannot be explained by methylation changes within these regions. The relationship between global DNA methylation and circadian clock gene expression therefore remains unclear. Given the use of high FA concentrations, a no-added-FA reference condition, and a single HNSCC cell model, these findings should be interpreted as preliminary. Further studies using additional HNSCC models, a broader range of FA concentrations, and mechanistic approaches are needed to clarify how FA exposure may influence epigenetic and circadian regulation in HNSCC.

## Figures and Tables

**Figure 1:**
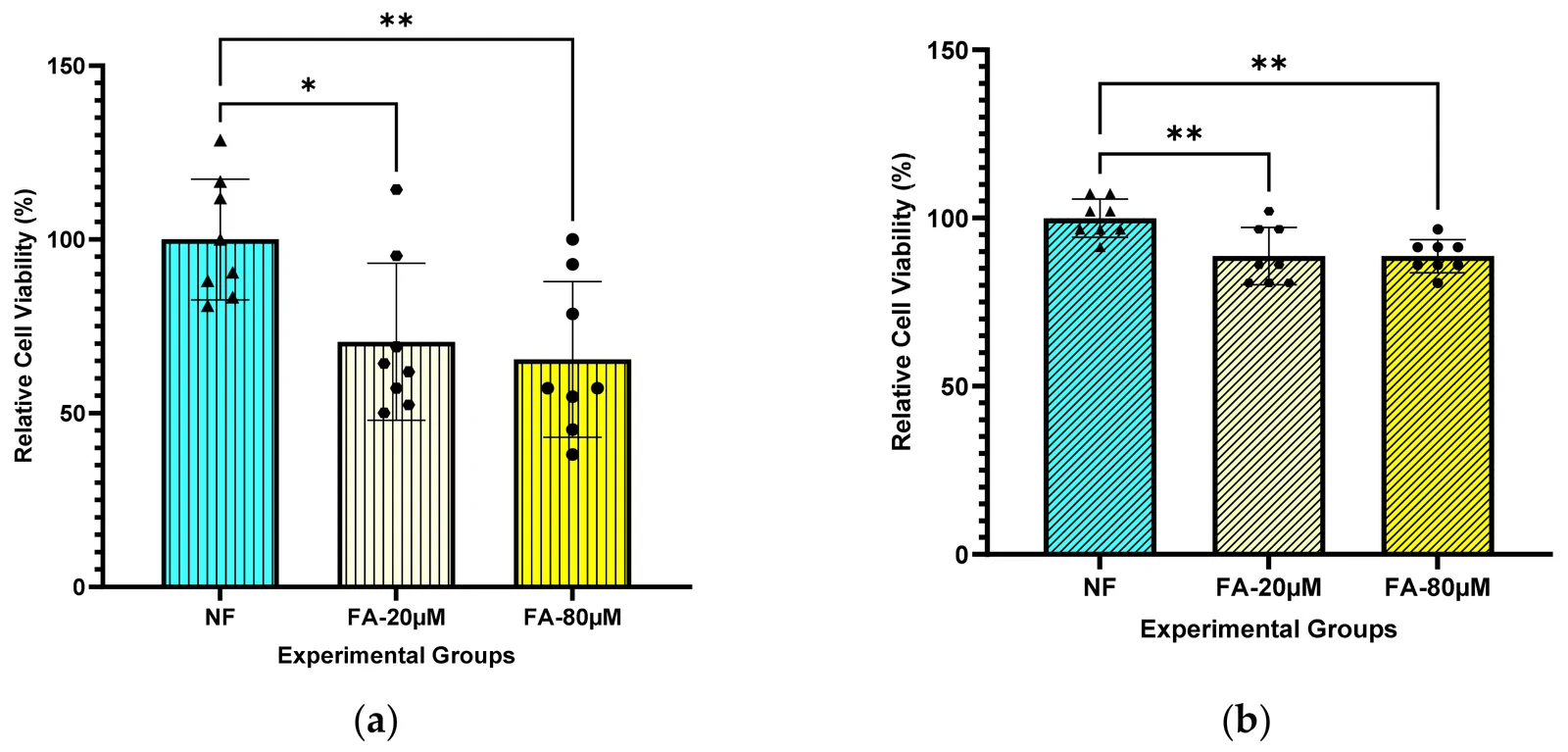
Effect of folic acid (FA) on FaDu cell viability: (**a**) cell viability after 48 h of FA treatment; (**b**) cell viability after 72 h of FA treatment. * *p* < 0.05 and ** *p* < 0.01 indicate statistical significance compared with NF.

**Figure 2:**
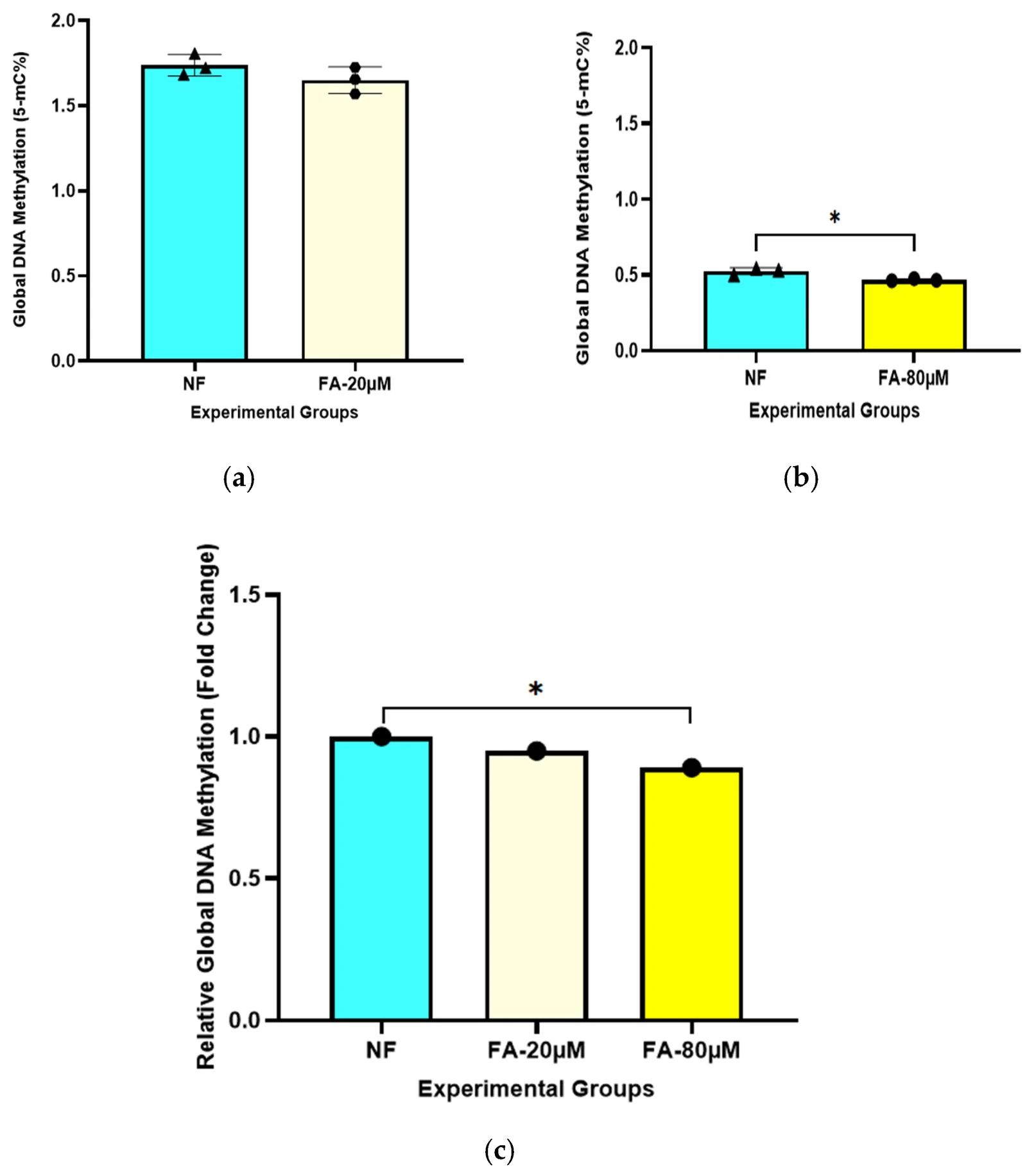
Effects of folic acid (FA) treatment on global DNA methylation in FaDu cells. (**a**) Global DNA methylation following treatment with FA-20 µM compared with the NF reference condition; (**b**) global DNA methylation following treatment with FA-80 µM compared with NF; and (**c**) relative global DNA methylation (fold change) following treatment with 20 and 80 µM FA, with NF set to 1. Data are presented as mean ± SD (*n* = 3 independent biological replicates). * *p* < 0.05 indicates statistical significance compared with NF.

**Figure 3:**
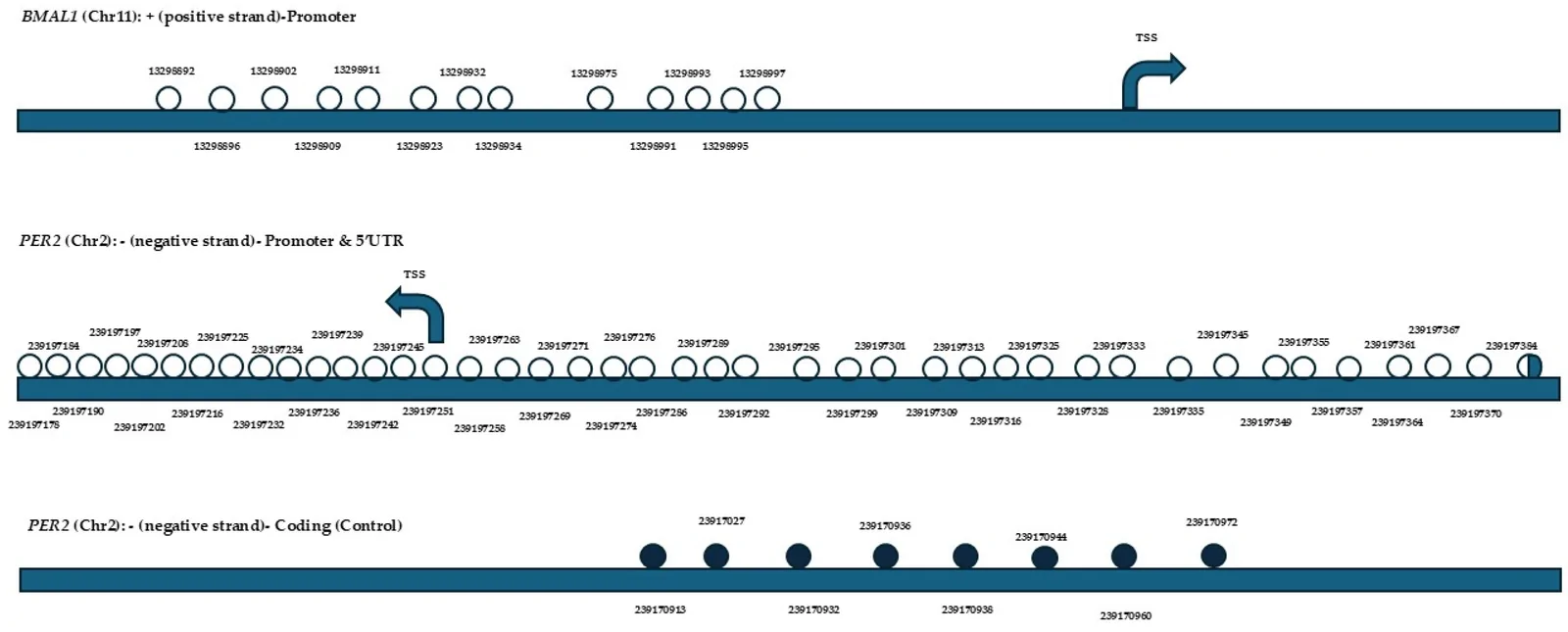
Methylation status of targeted CpG sites within *BMAL1* and *PER2*. Schematic representation of the CpG sites analyzed in the *BMAL1* promoter and *PER2* promoter/5′ untranslated region (5′ UTR), with genomic positions reported according to the human GRCh37 (hg19) genome assembly. Arrows indicate the transcription start site (TSS) and direction of transcription. Open circles represent unmethylated CpG sites, whereas filled circles represent methylated CpG sites, and the partially filled circle indicate a heterogeneous methylation pattern. No differences in methylation status at the analyzed CpG sites were observed between the NF and FA-treated groups. The *PER2* coding region was included as a methylated control.

**Figure 4:**
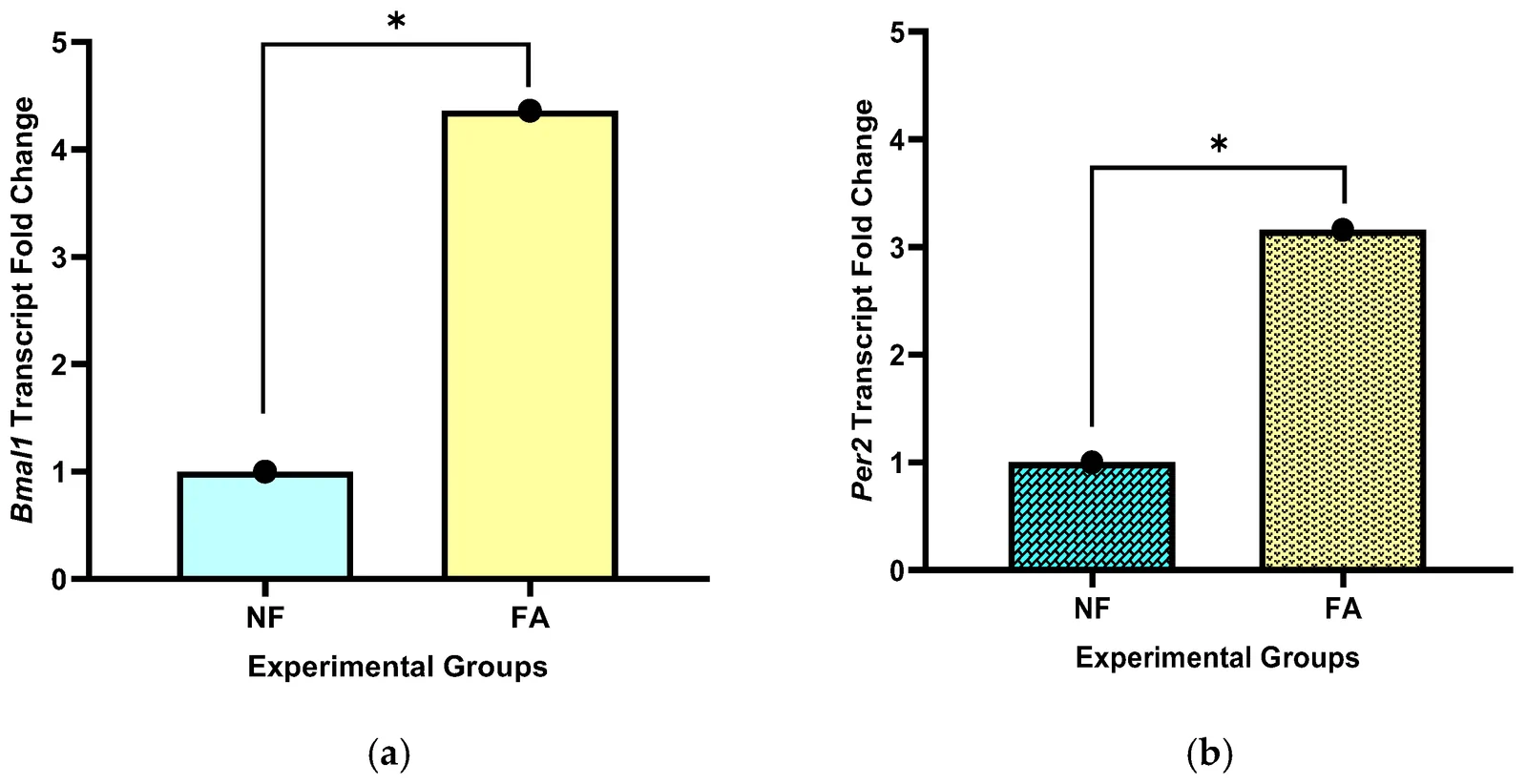
Folic Acid (FA) Upregulates *BMAL1* and *PER2* mRNA Expression in FaDu Cells: (**a**) *BMAL1* mRNA Expression; (**b**) *PER2* mRNA Expression. * *p* < 0.05 indicates statistical significance compared with NF.

**Figure 5:**
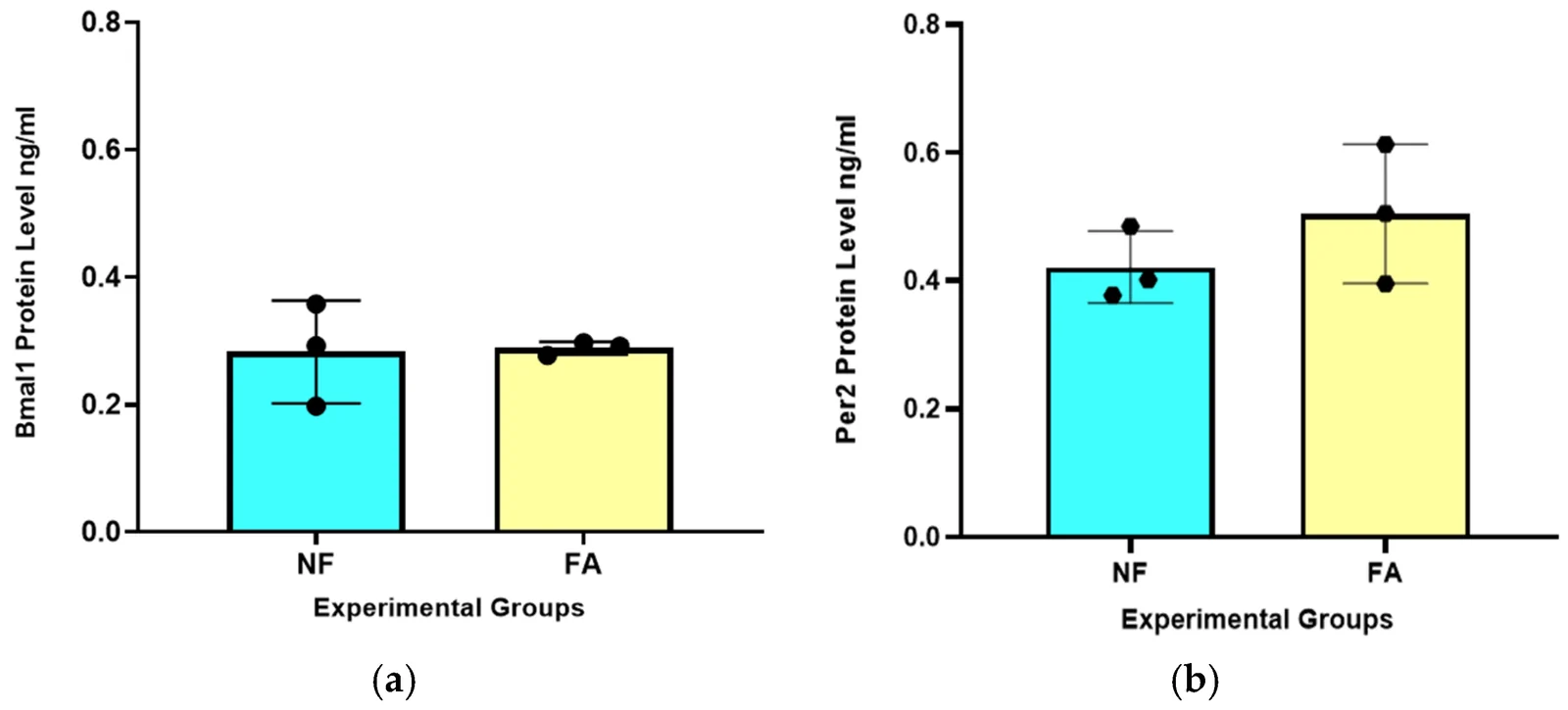
Protein expression in FaDu cells following folic acid (FA) treatment: (**a**) BMAL1 Protein Expression; (**b**) PER2 Protein Expression.

## Data Availability

The data supporting the findings of this study are included in the article and Supplementary Materials. Additional data are available from the corresponding author upon reasonable request.

## Supplementary Materials

The following supporting information can be downloaded at: https://www.mdpi.com/article/10.3390/nu18193181/s1, Supplementary Table S1. Absorbance values from the CCK-8 cell viability assay following 48 h of treatment with FA-20 µM and FA-80 µM in FaDu cells. Supplementary Table S2. Absorbance values from the CCK-8 cell viability assay following 72 h of treatment with FA-20 µM and FA-80 µM in FaDu cells. Supplementary Figure S1. Linear standard curve used for global DNA methylation quantification in the FA-20µM treatment experiment. Supplementary Figure S2. Linear standard curve used for global DNA methylation quantification in the FA-80µM FA treatment experiment. Supplementary Table S3. OD_450_ Measurements of 5-mC Standards Used for the FA-20 µM Global DNA Methylation Assay. Supplementary Table S4. OD_450_ Measurements for Global DNA Methylation (5-mC) in NF and FA-20 µM FaDu Cells. Supplementary Table S5. OD_450_ Measurements of 5-mC Standards Used for the FA-80 µM Global DNA Methylation Assay. Supplementary Table S6. OD_450_ Measurements for Global DNA Methylation (5-mC) in NF and FA-80 µM Folic FaDu Cells. Supplementary Table S7. Primer Details for Bisulfite- Targeted Amplification of Human Clock Genes. Supplementary Table S8. Genomic Coordinates and Target Sequences for Bisulfite Sanger Sequencing. Supplementary Table S9. Primer sequences for real-time qPCR analysis of human clock genes and the endogenous reference gene. Supplementary Table S10. qPCR Ct Ct Values for *BMAL1*, *PER2*, and *B2M* in NF and 80 µM Folic Acid-Treated FaDu Cells. Supplementary Figure S3. Four-parameter logistic standard curve used for PER2 protein quantification following treatment with FA-80 µM. Supplementary Table S11. OD_450_ Values of PER2 Protein Standards Used to Generate the Standard Curve for the ELISA Assay. Supplementary Table S12. OD_450_ Measurements of PER2 Protein in NF and FA-80 µM FaDu Cells. Supplementary Figure S4. Four-parameter logistic standard curve used for BMAL1 protein quantification following treatment with FA-80 µM. Supplementary Table S13. OD_450_ Values of BMAL1 Protein Standards Used to Generate the Standard Curve for the ELISA Assay. Supplementary Table S14. OD_450_ Measurements of BMAL1 Protein in NF and FA-80 µM FaDu Cells. Figure S5. Representative bisulfite Sanger sequencing chromatograms of CpG sites in the *BMAL1* and *PER2* genomic regions under NF and FA-80 µM conditions. (A) CpG sites within the *BMAL1* promoter region (CpG sites from c. 13298892 to c. 13298997, Chr11, positive strand), (B) CpG sites within the *PER2* promoter & 5′UTR region (CpG sites from c. 239197178 to c. 239197251, Chr2, negative strand), (C) CpG sites within the *PER2* promoter region (CpG sites from c. 239197258 to c. 239197384, Chr2, negative strand), (D) CpG sites within the *PER2* coding region (CpG sites from c. 239170913 to c. 239170972, Chr2, negative strand).

## Abbreviations

The following abbreviations are used in this manuscript:

FA	Folic Acid
cDNA	Complementary DNA
DNMTs	DNA methyltransferases
CpG	Cytosine-Phosphate-Guanine
HNSCC	Head and Neck Squamous Cell Carcinoma
NF	No-Added-FA
OSCC	Oral Squamous Cell Carcinoma
OD	Optical Density
PCR	Polymerase Chain Reaction
qPCR	Quantitative real-time PCR
UTR	Untranslated Region
