# Supplementary material for: Folic Acid Oversupplementation Alters Global DNA Methylation and Circadian Clock Gene Expression in FaDu Head and Neck Squamous Cell Carcinoma Cells: An Exploratory Study
Source: Nutrients. 2026 Sep 26;18(19):3181. doi: 10.3390/nu18193181 (PMC13666722; doi:10.3390/nu18193181)

Supplementary Material

Folic Acid Oversupplementation Alters Global DNA Methylation and Circadian Clock Gene Expression in FaDu Head and Neck Squamous Cell Carcinoma Cells: An Exploratory Study

Negin Faramarzi Garous [1], Liubov Lobanova [2], Silvana Papagerakis [3,4], Jessica R. L. Lieffers [1], Raed S. Said [3,5] and Petros Papagerakis [3,4,6]*

[1] College of Pharmacy and Nutrition, University of Saskatchewan, Saskatoon, SK S7N 5E5, Canada
[2] Department of Anatomy, Physiology and Pharmacology, College of Medicine, University of Saskatchewan, Saskatoon, SK S7N 5E5, Canada
[3] Department of Ophthalmology and Otorhinolaryngology–Head and Neck Surgery, Faculty of Medicine, Université Laval, Quebec City, QC G1V 0A6, Canada
[4] ARThrite Research Center, Université Laval, Quebec City, QC G1V 4G2, Canada
[5] Department of Anatomy, College of Medicine and Health Sciences (CMHS), United Arab Emirates University (UAEU), Al Ain, Abu Dhabi, P.O. Box 15551, United Arab Emirates
[6] Melbourne Dental School, University of Melbourne, Melbourne, VIC 3010, Australia
* Correspondence: petros.papagerakis@unimelb.edu.au

Supplementary Table S1. Absorbance values from the CCK-8 cell viability assay following 48 h of treatment with FA-20 μM and FA-80 μM in FaDu cells.

Biological Replicate	Replicate 1	Replicate 2
NF1	0.5483	0.5523
NF2	0.5126	0.5072
NF3	0.5583	0.5726
NF4	0.6547	0.6178
NF5	0.4136	0.4315
NF6	0.4235	0.4311
NF7	0.4905	0.4394
NF8	0.4631	0.4408
Blank	0.0274	0.0335

Biological Replicate	Replicate 1	Replicate 2
FA20-1	0.2512	0.2845
FA20-2	0.3702	0.3389
FA20-3	0.3215	0.2691
FA20-4	0.2989	0.2493
FA20-5	0.5802	0.571
FA20-6	0.4419	0.4869
FA20-7	0.3001	0.3201
FA20-8	0.3229	0.3454
Blank	0.0274	0.0335

Biological Replicate	Replicate 1	Replicate 2
FA80-1	0.4658	0.5325
FA80-2	0.2895	0.2655
FA80-3	0.3821	0.3988
FA80-4	0.3084	0.2786
FA80-5	0.2301	0.2623
FA80-6	0.4696	0.4272
FA80-7	0.3149	0.2685
FA80-8	0.1959	0.2224
Blank	0.0274	0.0335

Supplementary Table S2. Absorbance values from the CCK-8 cell viability assay following 72 h of treatment with FA-20 μM and FA-80 μM in FaDu cells.

Biological Replicate	Replicate 1	Replicate 2
NF1	0.2133	0.2107
NF2	0.2219	0.2021
NF3	0.2272	0.2371
NF4	0.2251	0.2384
NF5	0.2109	0.2297
NF6	0.2098	0.2328
NF7	0.2065	0.1972
NF8	0.2219	0.2018
Blank	0.027	0.028

Biological Replicate	Replicate 1	Replicate 2
FA20-1	0.2189	0.2241
FA20-2	0.2008	0.1821
FA20-3	0.1861	0.1969
FA20-4	0.1927	0.1706
FA20-5	0.2006	0.2225
FA20-6	0.1804	0.1821
FA20-7	0.1727	0.19005
FA20-8	0.2003	0.2206
Blank	0.027	0.028

Biological Replicate	Replicate 1	Replicate 2
FA80-1	0.2071	0.196
FA80-2	0.188	0.1948
FA80-3	0.2039	0.2178
FA80-4	0.1965	0.2063
FA80-5	0.1824	0.2004
FA80-6	0.1863	0.2167
FA80-7	0.1923	0.1706
FA80-8	0.1796	0.2032
Blank	0.027	0.028

Supplementary Figure S1. Linear standard curve used for global DNA methylation quantification in the FA-20μM treatment experiment.

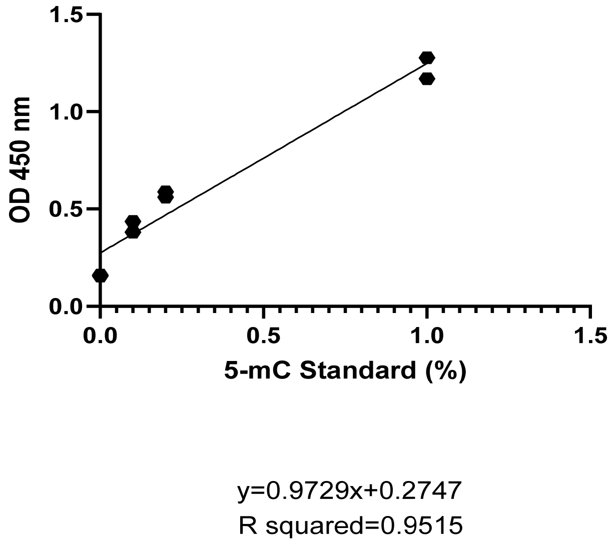

Supplementary Figure S2. Linear standard curve used for global DNA methylation quantification in the FA-80μM treatment experiment.

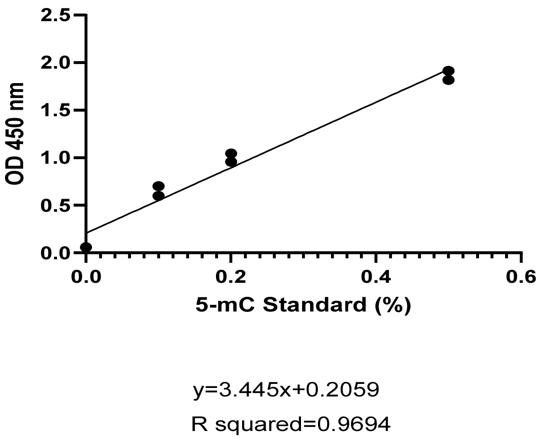

Supplementary Table S3. OD_{450} Measurements of 5-mC Standards Used for the FA-20 μM Global DNA Methylation Assay

	Replication 1	Replication 2	Replication 1	Replication 2	Replication 1	Replication 2	Replication 1	Replication 2	Replication 1	Replication 2	Replication 1	Replication 2
**5-mC Standard/Control (%)**	0 (NC)	0 (NC)	0.1	0.1	0.2	0.2	0.5	0.5	1	1	5	5
**OD_{450}**	0.156	0.161	0.380	0.435	0.588	0.561	1.371	1.141	1.169	1.277	2.388	2.489

Supplementary Table S4. OD_{450} Measurements for Global DNA Methylation (5-mC) in NF and FA-20 μM FaDu Cells

Biological Replicate	Replication 1	Replication 2
NF1	2.111	1.722
NF2	1.777	1.816
NF3	1.603	2.066
FA20-1	1.549	1.988
FA20-2	2.001	1.672
FA20-3	1.639	1.730

Supplementary Table S5. OD_{450} Measurements of 5-mC Standards Used for the FA-80 μM Global DNA Methylation Assay

	Replication 1	Replication 2	Replication 1	Replication 2	Replication 1	Replication 2	Replication 1	Replication 2	Replication 1	Replication 2	Replication 1	Replication 2
**5-mC Standard/Control (%)**	0 (NC)	0 (NC)	0.1	0.1	0.2	0.2	0.5	0.5	1	1	5	5
**OD_{450}**	0.059	0.066	0.701	0.599	1.044	0.958	1.914	1.818	1.975	1.896	2.351	2.295

Supplementary Table S6. OD_{450} Measurements for Global DNA Methylation (5-mC) in NF and FA-80 μM FaDu Cells

Biological Replicate	Replication 1	Replication 2
NF-1	1.867	1.917
NF-2	1.933	1.936
NF-3	1.972	1.599
FA80-1	1.429	1.875
FA80-2	1.739	1.589
FA80-3	1.738	1.663

Supplementary Table S7. Primer Details for Bisulfite-Targeted Amplification of Human Clock Genes

Gene	Primer Type	Sequence (5' → 3')
Human *BMAL1 (ARNTL)*	Forward	GGGAGTTAGGAATTGTTGTTTTT
Human *BMAL1 (ARNTL)*	Reverse	ATTTACCTACTACTTTCCTACCACCAAT
Human *PER2 Island 1-1*	Forward	TGTTTTTGGTTTTGTTTTAGGTG
Human *PER Island 1-1*	Reverse	CTAACCCTTAATTTCCTTAACCTTC
Human *PER2 Island 1-2*	Forward	GGGATAY*GTGTAGTTGTGGG
Human *PER2 Island 1-2*	Reverse	CTCAAACTCCTAAACTACR*AAAC

Note: * Indicates that 'Y' can represent either cytosine (C) or thymine (T), and 'R' can represent either adenine (A) or guanine (G).

Supplementary Table S8. Genomic Coordinates and Target Sequences for Bisulfite Sanger Sequencing

**Gene Name**	Human *BMAL1(ARNTL)* CpG Island 1	Human *PER2* CpG Island 1	Human *PER2* CpG Island 2	Human *PER2*
**Genomic Location (GRCh37)**	Chr11: + (positive strand)<br>13298890-13299004	Chr2: - (negative strand)<br>239197173-239197252	Chr2: - (negative strand)<br>239197253-239197390	Chr2: - (negative strand)<br>239170888-239170980
**Location**	Promoter	Promoter & 5′UTR	Promoter	Coding (Control)
**# of CpG**	13	16	28	8
**Genomic Target Sequence**	CCCGGGCGACCCCGAGGAGCGCGGC<br>TTGGGCACCGCAGTGGCCGCGGCTAG<br>TGGGAGACCTGAGGGGAAAGGGAGA<br>GGGCAGGGGCGAGGAACCCAGGGA<br>GCGCGCGCGGATTGG	ACGTAACCGCCGCCGGCGCGCGGGCC<br>TCGGGCAGGTCGGGGTCCCAGCGCCG<br>GCTCGGGCAGCGGAGGCGCCGCCGGA<br>AG	GGGATACGTGCAGCTGTGGGCGGCGG<br>CGGCGGGCGCGGGGCCGGGCGGACAG<br>AGCCGCGAGTCGGCGGAGGGACCGGC<br>GGACGGGCTGACGCGGGCGCGGCCGG<br>CGGTAAGTGGCGCGGCGCGGCCCCGC<br>TGCGGCTT	CAGTCAGGCGGGCAGCCTTTCGACT<br>ATTCTCCCATTCGGTTTCGCGCCCGG<br>AACGGAGAGTACATCACGTTGGACA<br>CCAGCTGGTCCAGCTTC
**Bisulfite Converted Target Sequence**	TTYGGGYGATTTYGAGGAGYGYGGTTTGG<br>GTATYGTAGTGGTYGYGGTTAGTGGGAGA<br>TTTGAGGGGAAAGGGAGAGGGTAGGGG<br>YGAGGAATTTAGGGAGYGYGYGYGGATT<br>GG	AYGTAATYGTYGTYGGYGYGYGGGTTTY<br>GGGTAGGTYGGGGTTTTAGYGTYGGTT<br>YGGGTAGYGGAGGYGTYGTYGGAAG	GGGATAYGTGTAGTTGTGGGYGGYGGY<br>GGYGGGYGYGGGGTYGGGYGGATAGA<br>GTYGYGAGTYGGYGGAGGGATYGGYG<br>GAYGGGTTGAYGYGGGYGYGGTYGGYG<br>GTAAGTGGYGYGGYGYGGTTTYGTTGY<br>GGTTT	TAGTTAGGYGGGTAGTTTTTYGATTAT<br>TTTTTTATTYGGTTTYGYGTTYGGAAY<br>GGAGAGTATATTAYGTTGGATATTAG<br>TTGGTTTAGTTTT

Supplementary Table S9. Primer sequences for real-time qPCR analysis of human clock genes and the endogenous reference gene.

**Primers**	**Forward primer (5′ – 3′)**	**Reverse primer (5′ – 3′)**
BMAL1	GACGATGAATTGAAACACCTCA	GAGTATCTTCCCTCGGTCAC
PER1	TGATTGCAGAGCGCATCCA	TGTGTGCCGCGTAGTGAAA
PER2	GCCTGATGATGGCAAAATCTGAA	GTGTGTGTCCACTTTCGAAGAC
B2M	TTAGCTGTGCTCGCGCTAC	CTCTGCTGGATGACGTGAGTAA

Supplementary Table S10. qPCR Ct Values for *BMAL1*, *PER2*, and *B2M* in NF and FA-80 µM FaDu Cells

	BMAL1		*PER2*		*B2M*	
**Biological Replicate**	**Replication 1**	**Replication 2**	**Replication 1**	**Replication 2**	**Replication 1**	**Replication 2**
NF1	19.94207	20.41451	23.95349	22.66486	13.50584	14.42452
NF2	20.19892	20.81034	22.37560	23.95245	12.99852	13.06981
NF3	20.88336	20.90572	23.90870	23.82650	13.28826	12.99266
FA1	17.39476	17.21935	22.41856	20.97493	12.70581	13.18811
FA2	18.14146	17.98902	21.24348	21.50278	12.98000	13.17143
FA3	17.99994	18.87981	21.23582	21.74268	13.72133	12.93095

Supplementary Figure S3. Four-parameter logistic standard curve used for PER2 protein quantification following treatment with FA-80 µM.

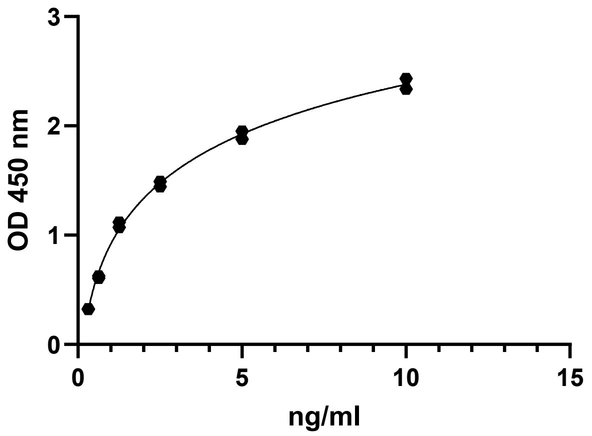

Supplementary Table S11. OD_{450} Values of PER2 Protein Standards Used to Generate the Standard Curve for the ELISA Assay

	Rep 1	Rep 2	Rep 1	Rep 2	Rep 1	Rep 2	Rep 1	Rep 2	Rep 1	Rep 2	Rep 1	Rep 2	Rep 1	Rep 2
**Standard ng/ml**	0 (NC)	0 (NC)	10	10	5	5	2.5	2.5	1.25	1.25	0.625	0.625	0.3125	0.3125
**OD_{450}**	0.146	0.16	2.586	2.849	2.031	2.103	1.597	1.645	1.223	1.271	0.778	0.759	0.48	0.473

Supplementary Table S12. OD_{450} Measurements of PER2 Protein in NF and FA-80 µM FaDu Cells

Biological Replicate	Replication 1	Replication 2
NF-1	0.686	0.669
NF-2	0.597	0.566
NF-3	0.541	0.561
FA-1	0.723	0.676
FA-2	0.813	0.789
FA-3	0.519	0.627

Supplementary Figure S4. Four-parameter logistic standard curve used for BMAL1 protein quantification following treatment with FA-80 μM.

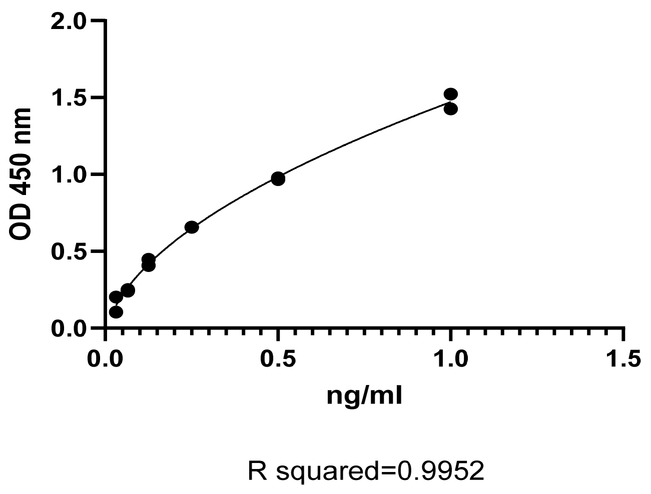

Supplementary Table S13. OD_{450} Values of BMAL1 Protein Standards Used to Generate the Standard Curve for the ELISA Assay

	Rep 1	Rep 2	Rep 1	Rep 2	Rep 1	Rep 2	Rep 1	Rep 2	Rep 1	Rep 2	Rep 1	Rep 2	Rep 1	Rep 2
**Standard ng/ml**	0 (NC)	0 (NC)	1	1	0.5	0.5	0.25	0.25	0.125	0.125	0.065	0.065	0.0312	0.0312
**OD_{450}**	0.064	0.072	1.591	1.494	1.045	1.033	0.724	0.726	0.476	0.516	0.318	0.309	0.27	0.172

Supplementary Table S14. OD_{450} Measurements of BMAL1 Protein in NF and FA-80 μM FaDu Cells

Biological Replicate	Replication 1	Replication 2
NF-1	0.626	0.627
NF-2	0.899	0.847
NF-3	0.805	0.756
FA-1	0.75	0.765
FA-2	0.767	0.807
FA-3	0.846	0.707

Supplementary Figure S5. Representative bisulfite Sanger sequencing chromatograms of CpG sites in the *BMAL1* and *PER2* genomic regions under NF and FA-80 μM conditions. (A) CpG sites within the *BMAL1* promoter region (CpG sites from 13298892 to 13298997, Chr11, positive strand), (B) CpG sites within the *PER2* promoter & 5'UTR region (CpG sites from 239197178 to 239197251, Chr2, negative strand), (C) CpG sites within the *PER2* promoter region (CpG sites from 239197258 to 239197384, Chr2, negative strand), (D) CpG sites within the *PER2* coding region (CpG sites from 239170913 to 239170972, Chr2, negative strand). For each panel, row (a) indicates the genomic reference sequence, row (b) shows the bisulfite-converted reference sequence, row (c) corresponds to the NF control group, and row (d) corresponds to the FA-80 μM group. Genomic positions are based on the Human GRCh37 reference genome.

A

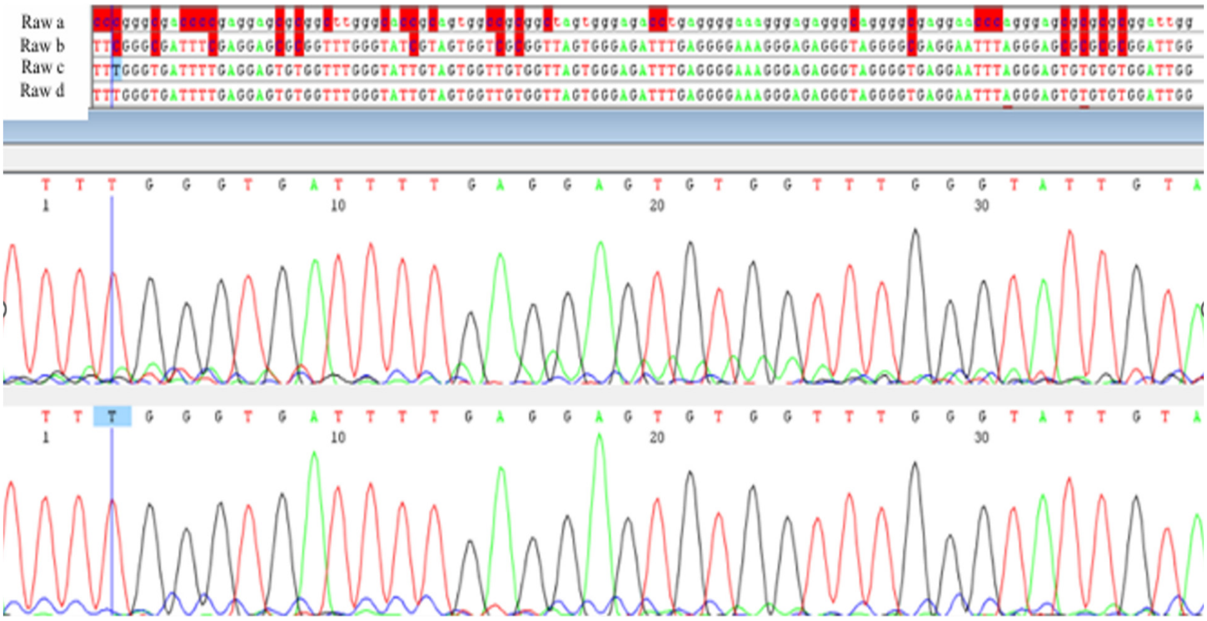

B

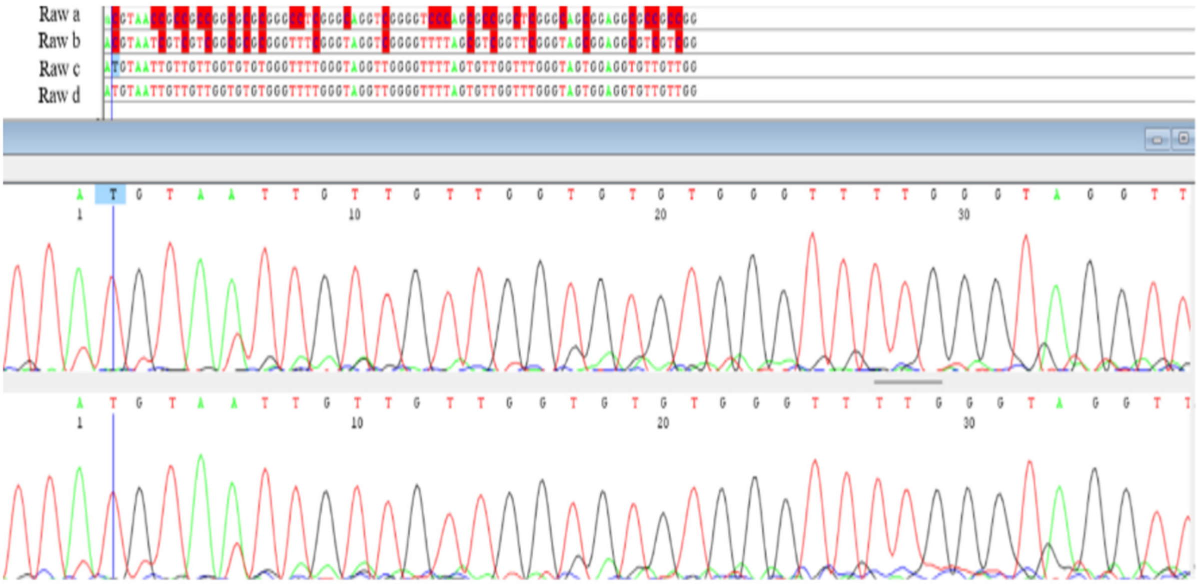

C

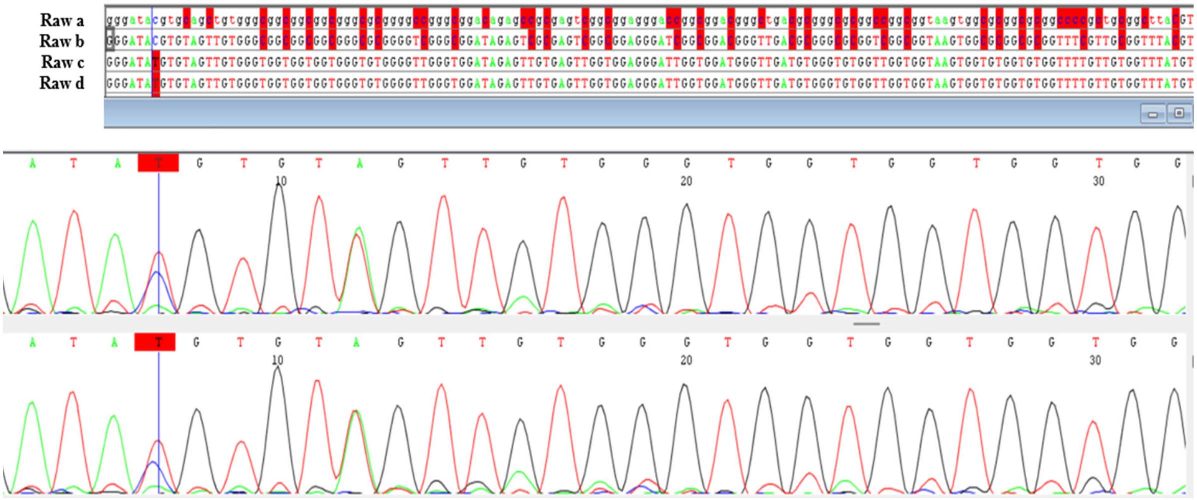

D

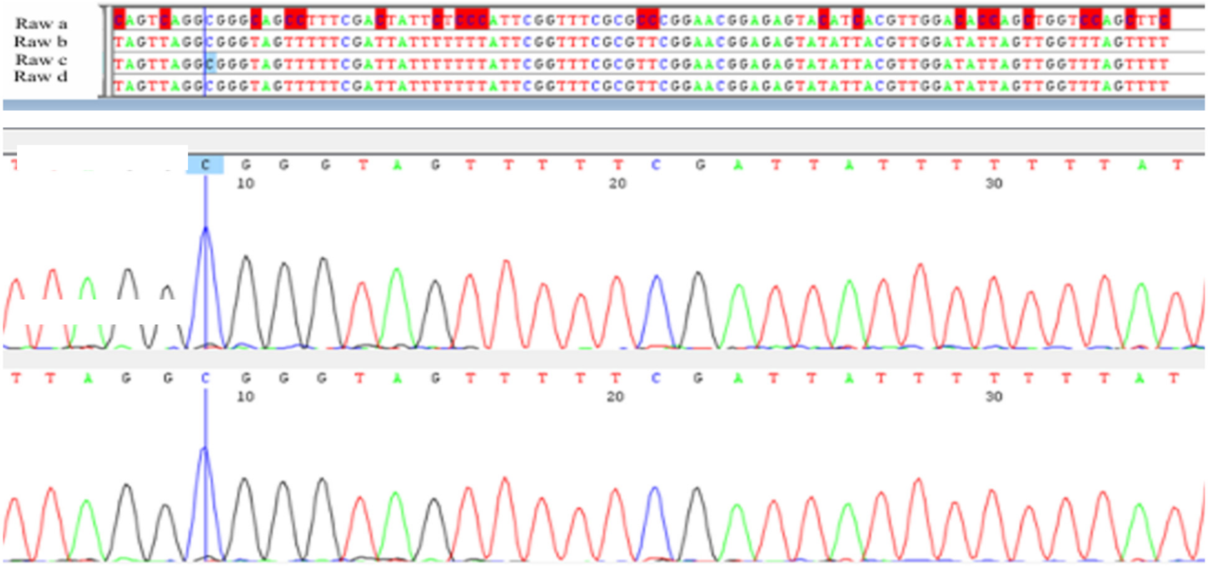

Supplement: Supplementary file 1 [file nutrients-18-03181-s001.zip › nutrients-4543570-supplementary.pdf]
